# The impact of the digital economy on the green transformation of the sports industry: the moderating role of marketization of data factors

**DOI:** 10.3389/fspor.2026.1728840

**Published:** 2026-02-06

**Authors:** Xinxin Zhang, Zhilei Cui, Liqun Jiang, Huimin Ding, Lu Zhong

**Affiliations:** 1 East China Normal University, Postdoctoral Research Station, Shanghai, China; 2 Myongji University, School of Physical Education, Yongin, Republic of Korea; 3 Yantai Nanshan University, School of Economics and Management, Yantai, China

**Keywords:** digital economy, fixed-effects model, green transformation, marketization of data factors, moderation effect model, sports industry

## Abstract

**Background:**

In the era of rapid digital technology advancement, the digital economy is profoundly reshaping industrial operation models, providing new momentum and development pathways for the green transformation of the sports industry.

**Objective:**

This study investigates how the digital economy facilitates structural optimization and explores the underlying mechanisms through which it drives the green and high-quality transformation of the sports industry.

**Methods:**

Using provincial-level panel data from China spanning 2015–2023, this study employs fixed-effects panel models and moderation effect models to empirically examine the relationship between the digital economy and the green transformation of the sports industry, while testing its internal mechanisms.

**Results:**

The empirical findings demonstrate that the digital economy exerts a significant positive effect on the green transformation of the sports industry. This conclusion remains robust after addressing potential endogeneity concerns and conducting multiple robustness checks. Furthermore, the marketization of data factors significantly strengthens the positive impact of the digital economy on the sports industry's green transformation. In terms of regional heterogeneity, the promotional effect of the digital economy is notably more pronounced in eastern regions than in central and western regions.

**Discussion:**

The results highlight that the digital economy acts as a key driver of the green transformation of the sports industry. Promoting the marketization of data factors can further amplify this effect. Policy implications include improving data property rights and trading systems, strengthening digital infrastructure and governance capabilities, and implementing differentiated regional strategies to achieve coordinated and sustainable upgrading of the sports industry.

## 1 Introduction

The 20th National Congress of the Communist Party of China emphasized its commitment to comprehensive transformation and reaffirmed the principle that “lucid waters and lush mountains are invaluable assets.” It further called for the integration of green transformation requirements into the overall economic and social development framework, promoting transformation across all dimensions, sectors, and regions, and fostering a harmonious coexistence between humanity and nature. As a green and low-carbon sunrise sector, the sports industry plays a vital role in stimulating domestic demand and acts as a new engine of economic growth. By 2023, the total scale (gross output) of China's sports industry reached 3.6741 trillion yuan, with an added value of 1.4915 trillion yuan, representing 1.15% of the national GDP. Since the inception of the 14th Five-Year Plan, China's sports industry has entered a new stage of high-quality development. Amidst ongoing economic transitions and emerging societal needs, it is imperative to address new development challenges, actively promote green innovation, and realize high-quality growth within the sports sector (1).

**Table 1:** Evaluation system for the green transformation of the sports industry.

Primary indicator	Secondary indicator	Indicator description	Attribute
Green transformation of industrial scale	Total Output of the Sports Industry	Total value of production activities in the sports industry	+
	Value Added of the Sports Industry	Value added generated by sports industry production activities	+
	Per Capita Sports Venue Area	Average area of sports venues per capita	+
	Number of Sports Venues	Total number of sports venues	+
	Total Employment in the Sports Industry	Total number of employees in the sports industry	+
	Per Capita Sports Park Green Area	Average green area of sports parks per capita	+
Green transformation of industrial structure	Proportion of Sports Industry Output	Share of total sports industry output in GDP	+
	Proportion of Sports Industry Value Added	Share of sports industry value added in GDP	+
	Proportion of Sports Fixed Investment	Share of sports industry fixed asset investment in total social investment	+
	Per Capita Sports Consumption Level	Share of per capita sports consumption in disposable income	+
	Economic Efficiency of Green Sports Industry	Contribution rate of the tertiary industry	+
Green transformation of industrial efficiency	Value-Added Rate of Sports Industry	Ratio of value added to total output	+
	Annual Growth Rate of Sports Industry Value Added	Year-on-year growth rate of sports industry value added	+
	Profit Margin of Sports Enterprises	Ratio of profit to total revenue of sports enterprises	+
	Total Factor Productivity (TFP)	Level of green total factor productivity	+

In the current era, the traditional production system—primarily dependent on capital, land, labor, and energy as input factors—can no longer sustain high-quality economic development. Therefore, promoting structural transformation and upgrading, as well as identifying and cultivating new drivers of economic growth, has become an inevitable strategic choice. The digital economy, emerging as a new economic paradigm following traditional agrarian and industrial economies, fundamentally reshapes and upgrades modes of production and consumption, serving as a crucial engine for China's high-quality economic development (2). Consequently, the digital economy functions as a fundamental pillar supporting the green transformation of the sports industry. On the one hand, it enhances production efficiency, optimizes resource allocation, reduces costs, and promotes technological advancement and industrial upgrading through green innovation. On the other hand, by advancing data analytics, intelligent technologies, and digital platforms, it facilitates business model innovation and international diffusion within the sports sector, enabling the virtualization and globalization of sports events.

Moreover, scientific and technological advancements, together with successive industrial revolutions, have become pivotal forces reshaping the global economic landscape, with the data-driven digital economy increasingly occupying a central position. The Three-Year Action Plan for Data Element X (2024–2026), jointly issued by China's National Bureau of Statistics and other relevant ministries, emphasizes enhancing synergy in data utilization to foster new industries, business models, and growth drivers, thereby providing strong support for advancing green development (3). Specifically, the marketization of data factors facilitates precise data analysis and intelligent decision-making, enabling a more accurate understanding of market demand and more efficient resource allocation, thereby accelerating the development of the digital economy. Against this backdrop, an important question arises: what impact does the digital economy exert on the green transformation of the sports industry, and through what mechanisms does this occur? Accordingly, this study constructs a benchmark regression model to examine the impact of the digital economy on the green transformation of the sports industry and its underlying mechanisms, thereby providing empirical insights and policy implications for the green transition of the sports sector.

## 2 Literature review

Research on the sports industry has made substantial progress under the dual influence of policy incentives and market demand, showing a diversified and systematic trajectory of development (4). In recent years, driven by national sports industry policies, scholars have increasingly emphasized green development in the sports sector, achieving fruitful research outcomes. At the macro level, scholars have examined green development within the sports industry. Song et al. (5) found that cultivating diversified consumption scenarios—such as the premier economy, winter sports economy, and silver economy—can promote the sustainable growth of emerging economic forms within the sports industry. Yu et al. (6) identified key opportunities for green and low-carbon development in the sports industry, including technological innovation leadership, smart technology-driven progress, model transformation, consumption concept guidance, and cross-sectoral integration. Xu et al. (7) suggested that integrating digital and physical elements accelerates structural transformation and upgrading, optimizes production factor allocation, facilitates green and low-carbon practices, and promotes the sustainable development of the sports industry. At the micro level, scholars have focused on market mechanisms and industrial chain coordination. Kang (8) argued that the institutional environment constitutes the foundational framework for the effective operation of the sports industry and should be strengthened through top-level design to establish a mutually reinforcing and supportive system. Zhang (9) found that factor supply and market demand are the two fundamental endogenous drivers promoting the green and high-quality development of the sports industry. Li (10) examined the integrated development of the digital economy and sports industry and found that their coupling degree remains highly correlated, with coordination evolving from near-critical to high-quality levels.

Currently, academic research on the digital economy has been expanding rapidly. Existing studies primarily investigate the impact of the digital economy on industrial green development from the perspectives of technological innovation, resource allocation, and institutional transformation. Jiali et al. (11) proposed that the digital economy, through the extensive application of technologies such as big data, artificial intelligence, and blockchain, promotes the informatization and intelligentization of production processes. This enhances both energy utilization efficiency and factor allocation efficiency, forming a green development pathway driven by technological innovation. Zhang et al. (12) argued that the digital economy reduces information asymmetry, optimizes resource flows across industrial chains, strengthens market-matching efficiency for green factors, and achieves “decarbonized growth” and “green spillover” effects. Yang (13) further argued that the proliferation of digital technologies reshapes corporate governance structures and regulatory models, promotes environmental information disclosure and green institutional innovation, and thereby establishes a new paradigm of data-empowered environmental governance.

Although both the digital economy and green industrial development have become prominent topics in contemporary policy and academic research, studies investigating the influence of the digital economy on the green development of the sports industry remain limited. Yan et al. (14) found that the digital economy promotes the free flow of sports production factors across regions, enhances digital collaboration within regional sports industries, and facilitates the realization of co-construction, co-governance, and shared benefits. Chen et al. (15) demonstrated that the digital economy exerts a positive effect on the green development of China's sporting goods manufacturing sector, primarily through three mechanisms: optimizing production factor allocation, innovating and upgrading industrial structure, and fostering leadership in digital governance.

In summary, the existing literature provides a solid theoretical foundation and empirical support for this study. Compared with previous studies, the main contributions of this paper are as follows: First, it theoretically elucidates the logical relationship between the digital economy and the green transformation of the sports industry, thereby enriching the research in related fields. Second, it introduces the marketization of data factors as a mediating variable and constructs a moderating effect model to further explore the mechanisms linking the digital economy and the green transformation of the sports industry, providing new perspectives for the green development of the sports sector. Third, it investigates regional heterogeneity by analyzing intercity differences in the digital economy and the green transformation of the sports industry, thereby offering plausible explanations for these disparities.

## 3 Theoretical analysis and research hypotheses

### 3.1 The impact of the digital economy on the green transformation of the sports industry

The rapid rise of the digital economy is reshaping the operational logic and development patterns of the sports industry, driving its green transformation through inherent systemic and evolutionary dynamics (16). At its core, the digital economy transcends a mere technological revolution; it constitutes a new economic paradigm centered on data as the fundamental production factor, supported by networked infrastructure and characterized by intelligent capabilities. By dismantling information barriers, improving resource allocation efficiency, and reshaping value creation mechanisms, it injects new momentum into the green development of the sports industry (17). Moreover, digital technologies enhance the transparency and measurability of sports production factors, enabling enterprises to achieve data-driven decision-making and optimized energy consumption, thereby reducing resource waste and carbon emissions. At the same time, the rise of digital platforms has facilitated the virtualization and servitization of sports consumption, shifting sports activities from high material dependence toward asset-light and low-energy operational models (18).

According to Sustainable Development Theory (19), the digital economy contributes to the rebalancing of the sports industry ecosystem. Its digital characteristics embed green principles throughout the entire lifecycle of the sports industry, reinforcing energy conservation and consumption reduction in production processes and encouraging low-carbon behaviors on the consumption side. More importantly, the openness and sustainability logic of the digital economy establish a new institutional foundation for constructing a green governance system in the sports industry, enabling enterprises to achieve a dynamic balance between environmental performance and economic efficiency.

In summary, the digital economy functions not only as a key driver of the green transformation of the sports industry but also as an intrinsic logical foundation for achieving high-quality and sustainable development within this sector. Accordingly, the following research hypothesis is proposed:

H1: The digital economy has a positive impact on the green transformation of the sports industry.

### 3.2 The moderating role of marketization of data factors in the impact of the digital economy on the green transformation of the sports industry

In the process by which the digital economy drives the green transformation of the sports industry, the marketization of data factors serves as a key catalytic mechanism. As an emerging production factor, the market-based allocation of data not only transforms the resource structure of the sports industry but also reshapes its mechanisms of value creation and transmission (20).

First, from the perspective of resource allocation, the marketization of data factors establishes mechanisms for defining data property rights, trading, pricing, and revenue distribution. This facilitates the efficient flow and optimized allocation of data resources, dismantling traditional barriers to factor mobility within the sports industry. Through data openness and sharing, sports enterprises can accurately identify consumers' green preferences and environmental responsibility expectations, thereby fostering green product innovation and low-carbon service provision (21). Second, from a technological empowerment perspective, the marketization of data factors provides institutional guarantees and incentive mechanisms for the deep application of digital technologies in the sports sector. Driven by the data element market, technologies such as artificial intelligence, cloud computing, and blockchain accelerate the intelligent and green transformation of the sports industry, making energy conservation, resource reuse, and sustainable operations attainable pathways (22). From an institutional and governance perspective, the marketization of data fosters multi-stakeholder collaborative governance mechanisms. Through the interaction among government regulation, platform facilitation, and corporate innovation, it promotes a dynamic equilibrium of green transformation within the digital economy.

Overall, the marketization of data factors acts not only as an moderating effect through which the digital economy affects the green transformation of the sports industry but also as a critical link for optimizing the industrial ecosystem and advancing high-quality development. The degree of marketization directly determines the depth and breadth of the digital economy's influence on the green transformation of the sports industry. Accordingly, the following research hypothesis is proposed:

H2: The marketization of data factors positively moderates the impact of the digital economy on the green transformation of the sports industry.

**Figure 1:**
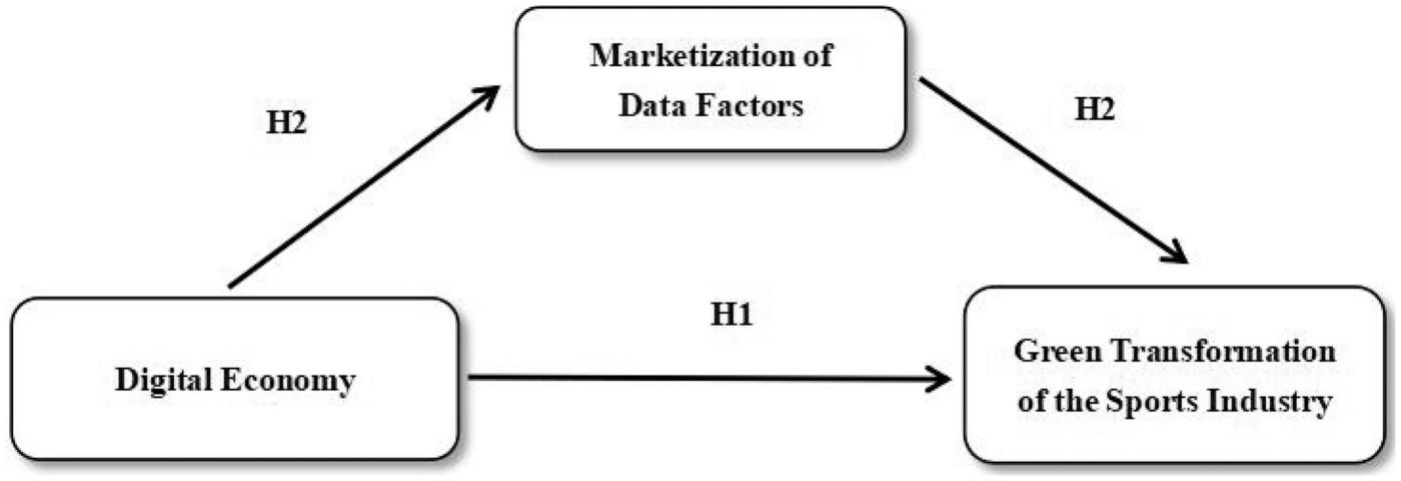
Path diagram of H1–H2 model.

In summary, according to the research hypothesis of this paper, the model path diagram of H1, H2 is constructed. As shown in Figure 1 below.

## 4 Research design

### 4.1 Variable selection

#### 4.1.1 The dependent variable in this study is the green transformation of the sports industry (*Gsi*)

The sports industry is a comprehensive sector centered on sports activities, encompassing multiple domains and stages. To measure its green transformation, this paper adopts the sports industry evaluation index system constructed by Li (23) which evaluates the transformation across three dimensions: scale, structure, and efficiency. Drawing on the methodologies proposed by Kuang (24) and Kang (25), 13 secondary indicators are established under these dimensions, as presented in Table 1. Following Zhang et al. (26), the entropy method is employed to determine the weights of these indicators. Each secondary indicator is first standardized using the extreme value method to obtain dimensionless values *x_ij_*. Then, the entropy method is applied to compute the comprehensive green transformation index of the sports industry for each region, as calculated by the following formula (Equations 1–4):

$$P_{ij}=\frac{X_{ij}}{\sum_{i=1}^{n}X_{ij}}$$ (1)

$$e_{j}=-\frac{1}{\ln n}\sum_{i=1}^{n}P_{ij}\;\ln(P_{ij})$$ (2)

$$g_{j}=1-e_{j}$$ (3)

$$w_{j}=\frac{g_{j}}{\sum_{\;j=1}^{m}g_{j}}$$ (4)

where *P_ij_* is the ratio of the indicator value for calculating the *i* evaluated object on the *j* evaluation indicator; *e_j_* is the entropy value of the *j* indicator, and *n* is the number of indicators; *g_j_* denotes the information entropy redundancy; and *w_j_* is the weight of the *j* indicator.

**Table 2:** Evaluation Index system of marketization of data factors.

Primary indicator	Secondary Indicator	Attribute
Marketization of data factors environment	Postal Network Service Area	+
	Total Number of Cable TV Network Users	+
	Number of IPv6 Allowable Access Users	+
Marketization of data factors development	Enterprise Technology Information Coverage Rate	+
	Number of Digital Industry R&D Institutions	+
	Number of Digital Industry Patent Applications	+
	Percentage of Total R&D Staff	+
Marketization of data factors scale	ICT Industry Revenue as a Percentage of Total Tax Revenue	+
	Total Industrial Output Value of the Digital Industry	+
	Percentage of Online Digital Service Users	+
Marketization of data factors management	Number of Employees in the Digital Industry	+
	Number of Legal Units in the Digital Industry	+
	R&D Investment Intensity	+

#### 4.1.2 Core explanatory variable: digital economy (*NQU*)

The digital economy constitutes a new economic paradigm characterized by data as a key production factor, digital technology as the primary driving force, and networked, intelligent operations as its defining features. It promotes the transformation of traditional industries toward higher efficiency and value restructuring. Following the methodology of Kou et al. (27), this study constructs a Digital Economy Index (*DE*) based on three dimensions: digital infrastructure, digital industrial development, and the digital economic environment. The index comprises three primary indicators, seven secondary indicators, and twenty tertiary measurement indicators. The entropy weighting method is employed to assign weights to each indicator, thereby minimizing subjective bias and enhancing the reliability of the constructed variable (*ibid*).

#### 4.1.3 Moderating variable: marketization of data factors (data)

##### 4.1.3.1 Marketization of data factors (data)

Compared with traditional factors, data as a production factor has virtual characteristics. Measuring it using a single evaluation indicator may lead to biased results. Therefore, drawing on the studies of Wang et al. (28) and Ma (29), this paper constructs an evaluation indicator system from four dimensions: the market-oriented environment of data factors, data development, data scale, and data management (Table 2). The entropy method is employed for calculation, and the specific calculation method is the same as above.

#### 4.1.4 Control variables

Control variables are incorporated to account for other factors that may affect the green transformation of the sports industry, thereby ensuring a more accurate estimation of the relationship between the digital economy and sports industry development. Drawing on existing literature, this study selects six control variables that potentially influence the development of the sports industry:

Social Consumption Level (*Lsc*): measured as the ratio of total retail sales of consumer goods to regional GDP, following Xiang (30);
Government Intervention Level (*Dgi*): expressed as the ratio of local general public budget expenditure to regional GDP, following Jiang (31);
Financial Development Level (*Fin*): following Xiang (26), measured as the ratio of year-end outstanding loans of financial institutions to GDP;
Foreign Direct Investment Level (*Fdi*): following Lan (32), measured as the ratio of annual FDI inflows to GDP;
Infrastructure Level (*Dlot*): following Gao (33), represented by the ratio of total highway mileage to regional land area;
Urbanization Level (*Urb*): following Kou (27), measured as the ratio of the urban population at year-end to the total resident population.

#### 4.1.5 Data sources

As the sports industry in several provinces developed relatively late and relevant data remain incomplete, most provincial-level administrative regions began releasing official statistical bulletins only after 2015. To ensure data authenticity, scientific rigor, and reliability, this study excludes the provinces and years lacking complete records. The study utilizes data from twenty-one provincial-level administrative regions in China—Beijing, Tianjin, Hebei, Shanxi, Inner Mongolia, Liaoning, Shanghai, Jiangsu, Zhejiang, Anhui, Fujian, Jiangxi, Shandong, Henan, Hubei, Hunan, Guangdong, Chongqing, Sichuan, Guizhou, and Yunnan—covering the period from 2015 to 2023, yielding a total of 189 observations. Missing data were supplemented through interpolation techniques. The data were obtained from multiple authoritative sources, including provincial sports bureaus, the National Bureau of Statistics, the official website of the General Administration of Sport of China, annual editions of the China Statistical Yearbook, and the China National Research Data Service (CNRDS).

### 4.2 Construction of the empirical model

To test Hypothesis 1 (H1), this study constructs Equation (5) to examine the impact of the digital economy (*Nqu*) on the green transformation (*Gsi*) of the sports industry. If H1 holds, the estimated coefficient (α1) of Nqu is expected to be significantly positive, indicating that the digital economy exerts a promoting effect on the green transformation of the sports industry. This result would confirm that the digital economy facilitates the green and sustainable development of the sports sector.

The model includes the selected control variables—social consumption level (Lsc), government intervention (*Dgi*), financial development (*Fin*), foreign direct investment (*Fdi*), infrastructure (*Dlot*), and urbanization (*Urb*). Additionally, Year represents the time fixed effect and City denotes the regional fixed effect, which control for the common influence of unobservable temporal and regional factors on sports industry development. *ε* represents the random error term.

$$Gsi_{it}=\alpha_{0}+\alpha_{1}Nqu_{it}+\alpha_{2}Ist_{it}+\alpha_{3}Dgi_{it}+\alpha_{4}Fin_{it}+a_{5}Fin_{it}+\alpha_{6}Dlot_{it}+\alpha_{7}Urb_{it}+Year_{t}+City_{i}+\varepsilon$$ (5)

To further test Hypothesis 2 (H2) regarding the moderating effect of Marketization of Data factors, Model (6) introduces an interaction term (*Nqu* × *Data*) to examine how marketization of data factors influences the relationship between the digital economy and the green transformation of the sports industry.

$$Gsi_{it}=\beta_{0}+\beta_{1}Nqu_{it}+\beta_{2}Nqu_{it}\times Data+\beta_{3}Data_{it}+\beta_{j}control_{it}+Year_{t}+City_{i}+\varepsilon_{it}$$ (6)

## 5 Mpirical results analysis

### 5.1 Descriptive statistical analysis

To better understand the variation characteristics of each variable and capture the developmental patterns across samples, descriptive statistical analysis was first performed for all variables. The results are reported in Table 3.

**Table 3:** Descriptive statistical analysis.

Variable name	Observations	Standard deviation	Minimum value	Maximum value	Mean
Digital economy	189	0.0861	0.0358	0.6173	0.133
green transformation of the sports industry	189	0.1948	0.0334	0.9019	0.2432
Marketization of data factors	189	0.0945	0.0003	0.6138	0.1180
Social consumption level	189	0.068	0.0029	0.5117	0.4048
Government intervention	189	0.0569	0.0944	0.3737	0.2082
Financial development level	189	0.4928	0.8318	3.379	1.5231
Foreign direct investment level	189	0.2584	0.0161	1.0919	0.3607
Infrastructure level	189	0.293	0.7054	1.9544	1.4416
Urbanization level	189	0.1189	0.4293	0.9377	0.6421

The digital economy variable has a mean of 0.133 and a standard deviation of 0.0861, suggesting limited dispersion and a relatively concentrated distribution among provinces. The mean value of the green transformation of the sports industry is 0.2432, with a standard deviation of 0.1948, indicating relatively high volatility and substantial regional and temporal heterogeneity. The marketization of data factors variable ranges from 0 to 1, where 1 indicates full marketization and 0 indicates the absence of marketization, reflecting pronounced inter-regional disparities.

For financial development, the relatively large standard deviation suggests significant regional disparities, which may be attributed to differences in local economic structures, financial policies, or resource endowments. Infrastructure and urbanization levels also display considerable variability, with infrastructure in particular showing a standard deviation of 0.293 and a maximum value approaching 2, indicating uneven levels of infrastructure investment across regions.

### 5.2 Benchmark regression analysis

To control for regional and time fixed effects, this study employs the green transformation of the sports industry as the dependent variable, while the digital economy serves as the core explanatory variable. Social consumption level, government intervention, financial development level, foreign direct investment level, infrastructure level, and urbanization level are included as control variables. A two-way fixed effects panel model was employed, and the results are reported in Table 4. *Gsi*(1) reports the benchmark regression results without control variables; *Gsi*(2) incorporates control variables but excludes regional and time fixed effects; and Model *Gsi*(3) includes both control variables and regional and time fixed effects. Across the three models, the estimated coefficients for the core explanatory variable—the digital economy (*Nqu*)—are 0.3501, 0.3281, and 0.2314, respectively. All are positive and statistically significant at the 1% level, indicating that, regardless of model specification, the digital economy exerts a robust and significant positive impact on the green transformation and development of the sports industry.

**Table 4:** Results of benchmark regression analysis.

Variable	*Gsi*(1)	*Gsi*(2)	*Gsi*(3)
*Nqu*	0.3501***	0.3281***	0.2314***
	2.53	2.28	2.08
*lsc*		0.1066	0.229
		0.55	1.31
*Dgi*		1.0945***	0.4069***
		3.64	1.65
*Fin*		−0.0602*	−0.0752*
		−1.25	−1.24
*Fdi*		0.0886*	0.0077**
		1.19	1.58
*Dlot*		−0.1065	−0.0758
		−1.77	−1.69
*Urb*		0.1884	0.3129
		0.83	0.88
*Year*	YES	NO	YES
*City*	YES	NO	YES
*R*2	0.4905	0.6841	0.4562
*F*	20.46***	103.38***	75.01***
*N*	189	189	189

The values in parentheses represent the t-statistic. ***, **, and * denote significance levels of 1%, 5%, and 10%, respectively. The same applies below.

These results reveal that, unlike traditional resource-driven growth models, the digital economy—centered on data—facilitates a dynamic equilibrium between high-quality development and green transformation in the sports industry through technological empowerment and efficient information flows. On the one hand, the widespread application of digital technologies enhances the transparency, intelligence, and precision of sports production and consumption activities. Energy and resource utilization can be monitored and optimized in real time, effectively reducing waste and emissions (32). On the other hand, the digital economy propels the sports industry's transformation from a material-intensive to a knowledge-intensive model, shifting growth drivers from factor-based to innovation-driven mechanisms. For instance, emerging sectors such as smart event management, virtual fitness, and online sports social networking not only meet diverse consumer demands but also reduce the carbon footprint of physical operations, thereby generating demonstration effects for green consumption. At the institutional level, the integration of digital governance and green finance further strengthens policy incentives and market constraints, fostering the accumulation of long-term competitiveness and guiding the sports industry toward a sustainable development trajectory.

Regarding the control variables, Models *Gsi*(2) and *Gsi*(3) yield regression coefficients for government intervention of 1.0945 and 0.4069, respectively, both statistically significant at the 1% level. This indicates that government intervention exerts a significant and positive influence on the green transformation of the sports industry. This effect likely arises because government intervention can effectively address market failures through policy support, infrastructure investment, and social welfare mechanisms. Furthermore, by regulating economic cycles and mitigating excessive fluctuations, governments help stabilize the socioeconomic environment, thereby fostering the green and sustainable development of the sports industry. The regression coefficients for foreign direct investment (*FDI*) are 0.0886 and 0.0077, respectively, indicating a significant positive effect. This positive effect may result from the role of international trade and foreign capital inflows, as an open market environment fosters competition, facilitates technological spillovers, and enhances the efficiency of green development within the sports industry.

By contrast, The regression coefficients of financial development are −0.0602 and −0.0752, suggesting a statistically significant inhibitory effect on the green development of the sports industry. This negative effect is not only attributable to the short-term profit–oriented nature of financial capital but may also be closely related to the bank-dominated structure of China's financial system (34). Credit allocation tends to favor traditional industries with stronger collateral bases and more stable cash flows, whereas green transformation projects in the sports industry are typically characterized by long investment horizons, high levels of uncertainty, and insufficient collateral, thereby limiting their access to financing. Moreover, under short-term performance evaluation mechanisms and risk-averse lending practices, financial institutions tend to reduce financing for green and innovative projects when economic uncertainty increases (35). In addition, existing green finance policies primarily focus on the energy and manufacturing sectors, providing limited support for the sports industry, which constrains the effective translation of financial deepening into long-term green investment in the sports industry.

Although social consumption, infrastructure, and urbanization constitute important pillars of economic development, they did not exhibit statistically significant effects in this analysis. This may suggest that these factors exert limited influence under the current economic structure or that other, more dominant determinants may be at play.

In summary, government intervention and foreign direct investment promote the green transformation of the sports industry, whereas the structural complexities of financial development produce inconsistent effects, highlighting the need for further optimization of financial policies and regulatory frameworks.

### 5.3 Robustness tests

#### 5.3.1 Endogeneity tests

Two potential sources of endogeneity may arise in examining the impact of the digital economy on the green transformation of the sports industry. First, the issue of omitted variables may exist: the influence of the digital economy could be affected by various unobservable regional or institutional factors, thereby introducing bias into the regression estimates. Second, a mutual causality (or reverse causality) problem may occur. On one hand, digital technologies enhance the dissemination, interactivity, and operational efficiency of sporting activities, thereby promoting industrial growth. On the other hand, the green development of the sports industry stimulates further innovation and application of digital technologies. Within this mutually reinforcing relationship, it is difficult to establish a clear causal direction between technological innovation and industrial transformation, suggesting a dynamic, bidirectional interaction mechanism.

To address potential endogeneity, this study employs the one-period lag of the digital economy variable (*Nqu-IV*) as an instrumental variable (*IV*) and applies the two-stage least squares (*2SLS*) estimation method. The results are presented in Table 5. In the first stage, the Kleibergen–Paap rk LM statistic yields a *P*-value below 0.01, rejecting the null hypothesis of under-identification. Additionally, both the Kleibergen–Paap rk Wald F-statistic and the Cragg–Donald Wald *F*-statistic exceed the Stock–Yogo critical value (15.87 at the 10% level), indicating that the instrumental variable is not weak and satisfies the relevance condition. In the second stage, after addressing endogeneity, the estimated coefficient of the digital economy remains positive and statistically significant at the 10% level, consistent with the benchmark regression results. These findings confirm that the empirical conclusions are robust and that the digital economy indeed exerts a significant and positive impact on the green transformation of the sports industry.

**Table 5:** Results of endogeneity test.

Variable	*Gsi*(4)	
	Phase I	Phase II
*Nqu*		0.1752***
		(1.52)
*Nqu-IV*	0.2331***	
	(2.12)	
*lsc*	0.1733	0.1578
	(1.57)	(1.13)
*Dgi*	0.1063**	0.0952***
	(1.95)	(2.57)
*Fin*	−0.0927*	−0.1027**
	(−2.64)	(−2.93)
*Fdi*	0.0197*	0.0558*
	(1.57)	(0.93)
*Dlot*	−0.2037	−0.1766
	(−1.67)	(−1.36)
*Urb*	0.0717*	0.1152
	(1.15)	(0.87)
*Year*	YES	YES
*City*	YES	YES
*R* ^2^	0.5079	0.4642
Phase I *F*-value	17.35***	
Kleibergen-paap rk LM	35.75***	
kleibergen-paap rk wald *F*	22.93*** [15.87]	
*N*	189	189

The values in square brackets represent the critical values for the Stock–Yogo test at the 10% significance level.

#### 5.3.2 Other robustness tests

##### 5.3.2.1 Truncation treatment

To further verify the robustness of the benchmark regression results and reduce the potential influence of extreme values, all continuous variables were subjected to winsorization at the upper and lower 1% quantiles. The regression model was then re-estimated using the adjusted dataset. As reported in Table 6[*Gsi*(5)], the regression coefficient of the digital economy variable remains significantly positive at the 1% level, confirming that the benchmark findings are not driven by outliers and that the main conclusions are robust.

**Table 6:** Results of robustness test.

Variable	*Gsi*(5)	*Gsi*(6)
*Nqu*	0.2275***	0.1578**
	(2.19)	(2.43)
*Control variable*	YES	YES
*Year*	YES	YES
*City*	YES	YES
*R* ^2^	0.6672	0.4758
*F*	18.72***	32.09***
*N*	189	153

##### 5.3.2.2 Exclusion of municipality samples

Municipalities directly under the central government differ substantially from other provinces in terms of economic structure, industrial composition, and policy environment, which may introduce bias or exert undue influence on the overall estimation results. To account for this, data from Beijing, Tianjin, Shanghai, and Chongqing were excluded, and the model was re-estimated using the remaining provincial-level samples. As shown in Table 6 (*Gsi*6), the coefficient of the digital economy remains significantly positive, indicating that the enabling effect of the digital economy on the green transformation of the sports industry is stable and robust even after removing these municipalities.

### 5.4 Mechanism analysis

#### 5.4.1 Moderating effect analysis

Moderating effect analysis examines how the strength or direction of a relationship changes under different conditions by introducing moderating variables, thereby enhancing the model's explanatory power. To evaluate whether marketization of data factors moderates the relationship between the digital economy and the green transformation of the sports industry, (Equation 6) was estimated. The coefficient of the interaction term reflects both the direction and significance of the moderating effect, with particular attention to the estimated coefficient of the interaction between marketization of data factors and the digital economy. Presented in Table 7, the results indicate that the regression coefficients of the digital economy and data factor marketization with respect to the green development of the sports industry are 0.2495 and 0.1739, respectively, and both coefficients are statistically significant at the 1% and 10% levels. When the two indicators are considered jointly, the coefficient of the interaction term is significantly positive at the 1% level, suggesting that marketization of data factors exerts a positive moderating effect on the relationship between the digital economy and the green transformation of the sports industry. Specifically, a higher level of data factor marketization strengthens the positive effect of the digital economy on the green development of the sports industry.

**Table 7:** Results of adjustment effect analysis.

Variable	*Gsi*(11)
*Nqu*	0.2495***
	(2.86)
*Data*	0.1739*
	(1.67)
*Nqu* × *Data*	0.2970***
	(3.04)
*Control variable*	YES
*Year*	YES
*City*	YES
*R^2^*	0.5069
*F*	28.6***
*N*	189

This finding suggests that, under the impetus of market-oriented data elements, institutionalized allocation mechanisms enhance the liquidity, accessibility, and utilization efficiency of data resources, thereby strengthening the penetration and enabling capacity of digital technologies in industrial green upgrading. On one hand, the refinement of data property rights confirmation, pricing, and trading systems facilitates the efficient aggregation and circulation of diverse data distributed across sports venues, event management, sports equipment manufacturing, and consumer behavior. This provides high-quality inputs for the application of artificial intelligence (*AI*), big data analytics, and the Internet of Things, thereby promoting substantial progress in energy efficiency optimization, carbon emission monitoring, and resource recycling (36).

On the other hand, the marketization of data factors reduces institutional frictions arising from information asymmetry and innovation diffusion barriers. This enables green consumption preferences to rapidly feed back to the industrial supply side, stimulating large-scale production and diversified innovation of green products and services. Consequently, a virtuous cycle of demand–supply interaction is established (37).

In summary, the marketization of data factors not only enhances the matching efficiency between the digital economy and the green transformation of the sports industry but also amplifies the synergistic effects of digital-driven green development through optimized resource allocation and cross-sectoral collaboration. This fosters the organic integration of technological innovation, industrial upgrading, and sustainable development.

### 5.5 Heterogeneity analysis

Significant differences exist across China's regions in terms of natural conditions, economic resources, and levels of development. Therefore, following the regional classification standard of the National Bureau of Statistics, this study divides the sample into three groups:Eastern Region: Beijing, Tianjin, Hebei, Liaoning, Shanghai, Jiangsu, Zhejiang, Fujian, Shandong, and Guangdong; Central Region: Shanxi, Inner Mongolia, Anhui, Jiangxi, Henan, Hubei, and Hunan; Western Region: Chongqing, Sichuan, Guizhou, and Yunnan. This classification enables the examination of regional heterogeneity in the effect of the digital economy on the green transformation of the sports industry.

As reported in Table 8, the regression results reveal pronounced regional disparities. The estimated coefficient of the digital economy variable in the Eastern Region is significantly higher than that in the Central and Western regions, suggesting that in more economically advanced and socially developed areas, the digital economy exerts a stronger positive effect on the green transformation of the sports industry.

**Table 8:** Heterogeneity test results.

Variable	*Gsi*	*Gsi*	*Gsi*
	Eastern	Central	Western
*Nqu*	0.2671***	0.1573*	0.1883*
	(2.91)	(2.07)	(1.61)
*Control variable*	YES	YES	YES
*Year*	YES	YES	YES
*City*	YES	YES	YES
*R* ^2^	0.0426	0.0555	0.5081
*F*	22.07***	40.26***	18.83***
*N*	90	63	36

These findings indicate that the role of the digital economy in promoting industrial green transformation is not a process of homogeneous diffusion, but rather a differentiated evolutionary mechanism shaped by regional development stages and institutional environments. The Eastern Region benefits from a high degree of technological penetration and resource integration in digital economic development. The advanced maturity of digital infrastructure, data factor markets, and innovation ecosystems allows digital technologies to be embedded more effectively along the sports industry value chain, thereby enabling green upgrading across production, operation, and consumption processes.

In contrast, the Central and Western Regions remain in the early stages of digital transformation. They exhibit relatively low efficiency in data resource allocation, limited technological spillover effects, and weaker innovation capabilities. Consequently, the digital economy's driving effect on the green transformation of the sports industry in these regions is comparatively weaker (38).

This regional heterogeneity highlights the nonlinear and uneven nature of digital economic development and its influence on green transformation. Therefore, future policy efforts should focus on reducing regional disparities in digital infrastructure, improving regional digital governance systems, and facilitating cross-regional data flow and resource sharing. Such measures would promote coordinated and balanced advancement of the sports industry's green transformation nationwide.

## 6 Discussion

First, the core findings of this study unequivocally confirm that the digital economy exerts a significant positive influence on the green transformation of the sports industry. This conclusion aligns with Hypothesis H1 and resonates with the central tenet of Sustainable Development Theory (19) which emphasizes technological innovation as a key driver of green growth. Through its distinct features of technological empowerment and efficient information flow, the digital economy alleviates traditional information asymmetries, enhances resource allocation efficiency, and fosters a multitude of low-carbon and green production and consumption models within the sports industry.

Specifically, digital technologies improve the transparency, traceability, and measurability of sports production factors. Enterprises can leverage big data analytics to achieve precise decision-making and optimize energy consumption, thereby reducing resource waste and carbon emissions (39). For example, intelligent event management systems enhance resource scheduling efficiency, while emerging models such as virtual fitness, online sports social platforms, and smart wearable ecosystems reduce dependence on physical resources, promoting environmentally friendly consumption patterns. These findings echo the perspectives of Yu Hao et al. (6), who emphasize that smart technologies and business model innovations are central to driving the green transformation of the sports industry. Collectively, this study reinforces the indispensable role of the digital economy as a productive force in advancing sustainable and low-carbon development across industrial systems.

Second, a key theoretical contribution of this study lies in its empirical validation that the marketization of data exerts a significant positive moderating effect between the digital economy and the green transformation of the sports industry, thereby supporting Hypothesis H2. This finding deepens our understanding of the enabling mechanisms of the digital economy and underscores the crucial role of data as a new production factor in amplifying digital-driven industrial transformation.

From a resource allocation perspective, the marketization of data promotes the efficient flow and optimal distribution of data resources by establishing institutional mechanisms for data property rights, transactions, pricing, and revenue sharing. These mechanisms dismantle structural barriers that traditionally restrict factor mobility within the sports industry (40). Consequently, sports enterprises can more accurately identify consumer preferences for green products and environmental responsibility, enabling agile innovation in green product design and low-carbon service provision (41). This mechanism aligns with Zhang Yinghao et al. (12), who argue that the digital economy enhances the matching efficiency of green factor markets by reducing information asymmetry and optimizing resource flows. From the technological empowerment perspective, the marketization of data factors provides both institutional guarantees and intrinsic incentives for the deep integration of artificial intelligence, cloud computing, and blockchain technologies within the sports sector. This integration accelerates the industry's shift toward intelligent and green transformation, consistent with Jiali Qian et al. (11) who highlight the role of digital technologies in improving energy efficiency and factor allocation. Moreover, these results reveal the powerful institutional driving force unleashed by data marketization as an emerging production factor in the digital economy. Therefore, this study expands the application boundaries of institutional economics within the context of digital economic transformation. It highlights the pivotal importance of well-defined data property rights, pricing mechanisms, and transaction rules in facilitating industrial green transformation. Collectively, the findings provide a more nuanced theoretical explanation for how the digital economy reshapes industrial upgrading pathways and accelerates sustainable development.

Third, this study identifies significant regional heterogeneity in the impact of the digital economy on the green transformation of the sports industry, with the eastern regions demonstrating particularly strong promotional effects. This finding carries important policy implications. As pioneers of China's digital economic development, eastern regions possess well-established digital infrastructure, highly active data element markets, and mature innovation ecosystems supported by abundant digital talent (42). Within this environment, digital technologies can more effectively penetrate all aspects of the sports industry, enabling the full realization and utilization of data element value and generating a stronger driving force for industrial green transformation.

In contrast, although the central and western regions also benefit from the digital economy, their relatively underdeveloped digital infrastructure, immature data markets, and structural limitations in industrial composition and human capital constrain the diffusion and spillover effects of digital technologies (43). As a result, the enabling role of the digital economy in promoting green transformation is comparatively weaker in these regions. Therefore, in advancing the nationwide green transformation of the sports industry, a uniform policy approach should be avoided. Instead, regionally differentiated policies and development strategies should be formulated based on each region's digital economic maturity, industrial foundation, and resource endowment. Such tailored approaches would foster coordinated and balanced development across regions and prevent widening disparities in digital-driven industrial transformation (44). Moreover, the Strategic Relocation Plan for Capital-, Technology-, and Labor-Intensive Industries, jointly issued by the CPC Central Committee and the State Council in September 2024, provides new opportunities for the green upgrading of the sports industry in central and western China (45). With appropriate national-level policy support, these regions can accelerate the adoption and application of digital technologies, strengthen the comprehensive green transformation of the sports industry, and enhance regional coordination and balance. Ultimately, this would contribute to a more inclusive, sustainable, and spatially balanced pattern of green economic development nationwide.

## 7 Conclusions

To investigate the impact of the digital economy on the green transformation of the sports industry, this study utilizes panel data from 21 Chinese provinces spanning 2015–2023. Employing fixed-effects models and moderation utility models, it analyzes the influence of the digital economy on the green transformation of the sports industry and explores its underlying mechanisms. The following conclusions are drawn: First, the digital economy exerts a highly significant promotional effect on the green transformation of the sports industry. Second, the marketization of data factors positively moderates the impact of the digital economy on this transformation. Third, regional heterogeneity exists in the digital economy's influence on the sports industry's green transformation, with eastern regions experiencing a more pronounced effect than central and western regions.

## 8 Limitations and future research directions

Despite yielding valuable insights, this study has certain limitations. First, in measuring marketization of data factors, the presence or absence of data trading platforms was used as a proxy variable, which may not fully capture the complexity and dynamism of marketization. Future research could develop more refined multidimensional indicators. Second, while the measurement framework for the sports industry's green transformation draws from existing research, there may still be room for optimization at certain micro levels. Additionally, this analysis primarily relies on provincial-level panel data. Future studies could utilize more granular firm-level data to explore the green transition pathways of different types of sports enterprises under the influence of the digital economy. Finally, this research focuses solely on the moderating effect of marketization of data factors. Future investigations could examine other potential moderating variables or mediating mechanisms, such as digital governance levels and green finance development, to construct more comprehensive and sophisticated theoretical models.

Looking ahead, as the digital economy continues to evolve and data factor markets mature, the sports industry's green transformation will encounter broader opportunities. Key future research directions include examining how cutting-edge technologies like artificial intelligence and blockchain can further optimize resource allocation and environmental performance within the sports industry under the digital economy, and exploring strategies to build a more resilient and sustainable green sports industry ecosystem.

## Data Availability

The datasets presented in this study can be found in online repositories. The names of the repository/repositories and accession number(s) can be found in the article/Supplementary Material.
